# Developmental Differences in Circular RNA Expression Between Adult and Fetal Human Salivary Glands Based on Public Total RNA-Sequencing Data

**DOI:** 10.3390/ijms27083608

**Published:** 2026-04-18

**Authors:** Zahra A. Aldawood, Alawi Habara

**Affiliations:** 1 Department of Biomedical Dental Science, College of Dentistry, Imam Abdulrahman Bin Faisal University, Dammam 34212, Saudi Arabia; 2 Department of Biochemistry, College of Medicine, Imam Abdulrahman Bin Faisal University, Dammam 34212, Saudi Arabia

**Keywords:** adult salivary glands, fetal salivary glands, circRNA, miRNA, human development of salivary glands

## Abstract

Circular RNAs (circRNAs) are stable regulatory RNAs whose developmental patterns in human salivary glands remain poorly defined. Publicly available total RNA-seq data from adult and fetal salivary glands (GSE143702—adult, n = 13; fetal, n = 14) were analyzed to profile the circRNA expression and evaluate developmental-stage differences. Reads were aligned with STAR using chimeric detection, circRNAs were parsed and annotated with CIRCexplorer2, and circRNAs supported by ≥2 back-splice junction reads were retained for quantification. Principal component analysis (PCA) of circRNA expression profiles demonstrated significant (PERMANOVA *p* = 0.001) separation between adult and fetal salivary glands, with a moderate effect size (R^2^ = 0.118). Differential expression analysis identified 18 circRNAs that were significantly (adjusted *p* < 0.05) upregulated in adult salivary glands, with three additional circRNAs showing evidence suggestive of differential expression (0.05 ≤ adjusted *p* < 0.10). In fetal salivary glands, 18 circRNAs were significantly upregulated, with eight additional circRNAs showing suggestive evidence. For functional context, stage-associated circRNAs were linked to predicted miRNA interactions using the circAtlas 3.0 database and then to experimentally supported miRNA target genes using the miRTarBase database. These findings provide a stage-resolved overview of salivary gland circRNAs throughout development and aid in the prioritization of candidates for downstream validation.

## 1. Introduction

Salivary glands are exocrine glands that produce and secrete saliva [1,2], which is essential for maintenance of oral health and the microbiota associated with it [3]. Saliva has numerous functions, including in lubrication and digestion, as an antimicrobial agent, and mineral protection in teeth [3,4]. In humans, approximately 90% of saliva is produced by the three major salivary glands (the parotid, submandibular, and sublingual glands), while the remaining 10% is produced by hundreds of minor salivary glands scattered throughout the oral cavity [5,6]. Valstar and colleagues describe an overlooked pair of salivary glands termed tubarial glands, which are in the nasopharynx near the eustachian tube [7]; however, their characterization has sparked debates regarding whether they are merely minor salivary glands [8,9]. Salivary glands can be affected by a broad range of pathologies, such as infectious, neoplastic, immune-mediated, and iatrogenic (radiation- or drug-induced) diseases [3,10,11,12,13,14]. Whatever the etiology of salivary gland disorders, dysfunctions considerably affect quality of life [15,16,17]. Unfortunately, the current therapies primarily provide only symptomatic alleviation in many cases [18,19]. 

Salivary glands are composed of heterogeneous populations of cells, including acinar, ductal, myoepithelial, neuronal, lymphatic, and endothelial cells [20]. Salivary gland development in humans begins during the sixth and seventh weeks of intrauterine life and involves complex processes [13], including epithelial bud formation, followed by branching morphogenesis, ductal elongation, and secretory cell differentiation [21]. Ultimately, this process leads to the formation of a well-structured gland with a well-designed hierarchy, improving the absorption and secretion efficiency by increasing the surface area [22].

Diverse signaling pathways that regulate different cellular processes interact to regulate the development, growth, and function of salivary glands [2], and dysregulation of these pathways can lead to dysfunction of the salivary gland and/or the progression of diseases [23]. For example, the FGF signaling pathway, along with its receptors and ligands, is critical for early salivary gland formation and the branching morphogenesis of salivary glands and other organs [24]. NOTCH signaling, a highly conserved cell signaling system that regulates cell fate determination during various developmental processes and regeneration, is activated during salivary gland development [1,2]. At different developmental stages, various NOTCH components are expressed to influence the epithelial cell fate of the developing buds and/or ducts, guide the differentiation of myoepithelial cells, and affect innervation through the parasympathetic system [25,26]. TGFβ signaling plays a critical role in organ development and morphogenesis across various tissues [27]. Similarly, various TGFβ components are expressed at different developmental stages to influence duct and acinar formation and branching [2,28,29].

Recent advancements in omics technologies have greatly improved our understanding of salivary gland biology via the generation of publicly accessible, comprehensive datasets [23,30,31,32,33,34], and these efforts have helped shape the field of salivary glands in health and disease [2,23,30,31,32,33,34]. Saitou et al. performed a transcriptomic analysis of major adult and fetal salivary gland tissues, integrating these data with publicly available proteomic data [34]. They established a transcriptomic landscape of healthy human salivary glands and found that regulation likely occurs at the transcriptional and post-translational levels [34]. Unsurprisingly, the transcriptomes for mature salivary glands showed the upregulation of pathways for secretory and immune functions, while fetal tissues were enriched with developmental genes [34]. In a recent study, computational analysis of single-cell RNA-sequencing (scRNA-seq) data from fetal submandibular glands across three gestational windows (12–13, 14–16, and 17–19 weeks) was used to characterize the cellular heterogeneity and map the dynamics of cell fate decisions [31]. In another study, the researchers combined RNA sequencing (RNA-seq) and scRNA-seq to foster a deeper understanding of human submandibular gland biology by analyzing healthy tissues from the submandibular glands of ten male and five female adult patients [32].

The study of salivary glands during the postnatal period is intriguing due to the significant impact of environmental factors [3]. Nonetheless, progress in this area remains limited by the scarcity of available tissue samples [35]. Fetal salivary gland tissues are typically obtained during elective abortions [31,34]. However, obtaining samples from neonates and children poses greater challenges due to ethical constraints that preclude invasive sampling [35]. Adult tissue samples are typically obtained during surgeries unrelated to the salivary gland, imposing additional limitations on this type of experiment [34]. Existing human studies on postnatal salivary gland development and function are limited, with most analyses based on RNA isolated from saliva rather than from gland tissue [36,37,38,39]. A previous study explored the salivary transcriptomics among neonates in relation to their ability to feed by mouth [35]. While this approach overcomes the ethical challenges of obtaining samples from neonates and children, it introduces complexity in data interpretation, as the molecular content of saliva reflects contributions from other tissues, such as lacrimal glands, gingival crevicular fluid, and, not to forget, the oral microbiota, desquamated mucosal cells, and other debris. This mixed origin contributes to the difficulty of targeting transcriptome analysis to specific cellular sources within the salivary glands.

Given the heterogeneity of cellular components and the complexity of the molecular mechanisms involved in fetal and adult salivary gland development and functions, there remains a significant gap in our understanding of the roles that circRNAs play in these processes. Therefore, it is essential to comprehensively characterize circRNA expression patterns in both fetal and adult salivary glands and elucidate their circRNA-mediated regulatory networks.

CircRNA is a special type of RNA molecule that is fundamentally different from linear RNAs, which are studied more often. In comparison with linear RNAs, which have 5′ and 3′ ends, circRNAs appear in covalently closed, continuous loops with no free ends [40,41]. This circularity makes circRNAs highly stable and exonuclease-resistant, which renders them more stable in the cell and plasma than linear RNAs [41,42]. CircRNAs were initially thought to be low-abundance splicing errors; however, recent studies have shown that they are widespread and evolutionarily conserved and exhibit tissue- and cell-specific expression patterns [43]. Moreover, circRNAs are recognized as significant controllers of gene expression, with various cellular roles, and will soon be exploited as critical disease markers.

CircRNAs are produced by a process known as back-splicing, wherein a downstream 5′ splice donor site is fused with an upstream 3′ splice acceptor site. This process is controlled by cis-acting elements in the RNA in the form of sequences of inverted repeats and trans-acting elements, such as RNA binding and protein factor splicing [43]. The efficiency of back-splicing depends on the existence of complementary sequences around the exons and the secondary structure of pre-mRNA [42]. CircRNAs can be categorized into three types: ecircRNAs (produced from exons), ciRNAs (produced from introns), and eiciRNAs (produced from exons and introns). CircRNAs are commonly more abundant in the cytoplasm; however, a portion of circRNAs stay in the nucleus, where they can control transcription or alter alternative splicing [44]. This circular arrangement gives circRNAs degradation resistance properties and thereby allows them to accumulate to noticeable levels even when their linear counterparts are expressed temporarily. The abundance of circRNAs is different when comparing tissues and developmental stages, and they are often highly expressed in both neural and exocrine tissues, indicating a possible tissue-specific role [41]. The functions of circRNAs are often linked to subcellular localization: cytoplasmic circRNAs commonly modulate post-transcriptional regulation through interactions with miRNAs and RNA-binding proteins, whereas nuclear circRNAs can influence gene expression by interacting with the transcriptional machinery and splicing factors [40,44,45].

Direct investigations of circRNAs in human salivary glands are rare. However, an analysis conducted on a goat model has shown that circRNAs are expressed differently in the submandibular glands throughout development and are also involved in immune regulation [46]. Salivary gland circRNAs are also in a position to mediate immune-related genes and circuits, such as natural killer cell-mediated cytotoxicity and T-cell receptor signaling, that play key roles in glandular immune responses [46].

CircRNAs of salivary glands stand out as the most interesting topic of study because these organs perform secretory, immune, and developmental functions. CircRNAs have the potential to regulate transcription, protein activity, and extracellular signaling, and they are considered a stable and tissue-specific salivary biomarker and therapeutic intervention candidate [41].

However, the available studies remain largely preliminary, and most of the corresponding functional knowledge is derived from other organs or animal models. Additionally, the original publicly available salivary gland tissue RNA-seq cohort study explores conventional linear gene expression and does not include circRNA-specific detection and quantification [34]. Because the original study was published in 2020, it predates the release of circAtlas 3.0, which became available in early 2024 and now enables circRNA-focused functional inference analysis. The key questions we address in this study are whether circRNA expression differs between adult and fetal salivary glands, and which circRNAs are most strongly associated with different developmental stages, addressing a current gap in the literature on circRNA profiles across salivary gland development. Therefore, we performed an adult vs. fetal salivary gland total RNA-seq analysis using publicly available data to profile the circRNA expression, compare gland- and stage-specific patterns, and characterize associated biological pathways using Reactome enrichment analyses. We also report circRNAs in this paper according to their circAtlas and uniform IDs to facilitate downstream reuse in future studies.

## 2. Results

### 2.1. circRNA Detection and Quantification

**Figure 1:**
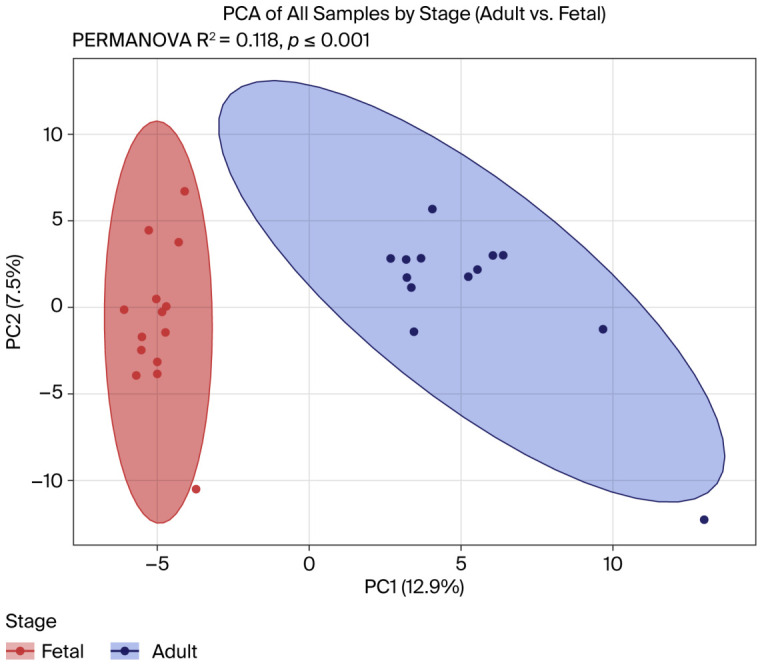
Principal component analysis (PCA) of circRNA expression profiles for adult vs. fetal salivary glands. Each point represents an individual sample (blue, adult; red, fetal). The PCA shows clear separation between the two groups, with a moderate effective size. The degree of group separation was confirmed to be statistically significant via PERMANOVA (R^2^ = 0.118, *p* = 0.001).

Total salivary gland tissue RNA-seq data were obtained from GEO (GSE143702). Fourteen and thirteen samples were obtained from adult and fetal salivary glands, respectively. The numbers of circRNAs detected for the individual samples (≥2 supporting reads) are summarized in Table S1. Statistical comparison revealed a significant difference in the circRNA counts between the adult and fetal salivary glands (Wilcoxon *p* = 0.044; Table S2), and the principal component analysis (PCA) of the circRNA expression (Figure 1) showed a statistically significant separation between adult and fetal salivary glands, with a moderate effective size (PERMANOVA R^2^ = 0.118, *p* = 0.001).

### 2.2. circRNA Differential Expressions Between Adult and Fetal Salivary Glands

**Figure 2:**
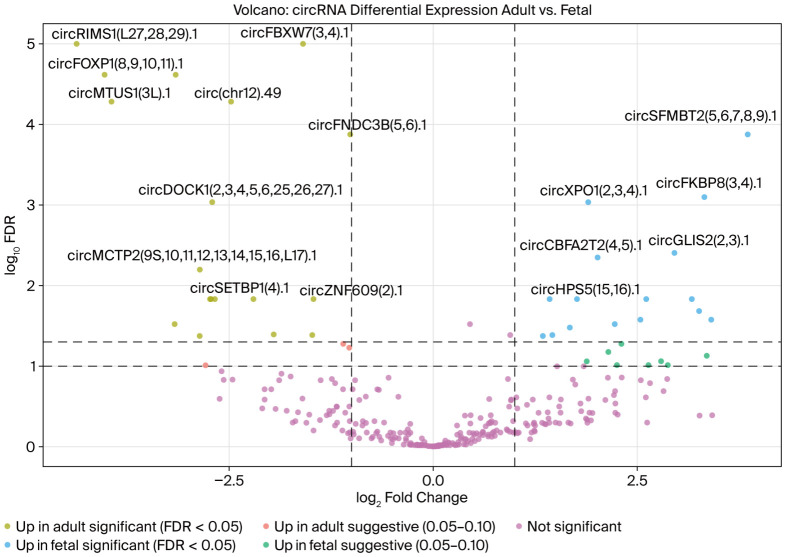
A volcano plot of circRNA differential expressions between adult and fetal salivary glands. Each point represents a circRNA, plotted as a log2-fold change (adult vs. fetal salivary glands) on the *x*-axis and as a log10 FDR (adjusted *p*-value) on the *y*-axis. Significant (adjusted *p*-values ≤ 0.05) and suggestive (adjusted *p*-values > 0.05 and <0.10) circRNAs are shown. CircRNAs are color-coded as follows: significantly upregulated in adult salivary glands in yellow; suggestive of upregulation in adult salivary glands in red; significantly upregulated in fetal salivary glands in blue; suggestive of upregulation in fetal salivary glands in green; not significant in purple. The differential expression analysis identified 18 significant and three suggestive circRNAs upregulated in the adult samples and 18 significant and eight suggestive circRNAs upregulated in the fetal samples.

The differential expression analysis of the circRNA profiles between adult and fetal salivary glands revealed that 18 significant and three suggestive circRNAs were upregulated in the adult salivary glands (Figure 2 and Table 1) and 18 significant and eight suggestive circRNAs were upregulated in the fetal salivary glands (Figure 2 and Table 2).

**Figure 3:**
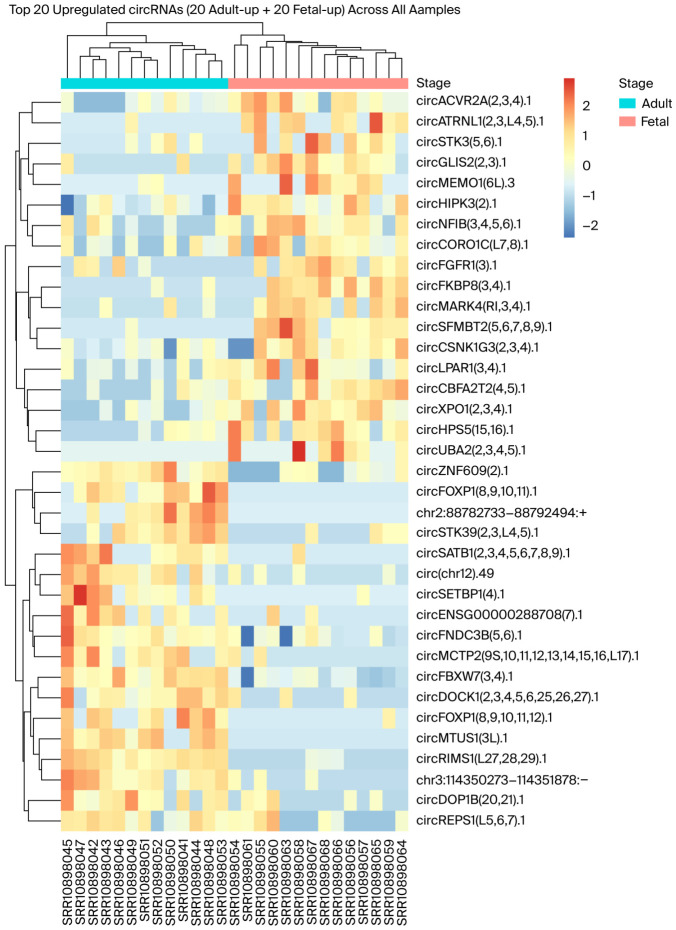
A heatmap of the top 20 differentially upregulated circRNAs across the adult and fetal salivary gland samples. Values represent row-scaled normalized expression (z-scores), where warmer (red) and cooler (blue) colors indicate higher and lower relative expressions, respectively. Samples (columns) and circRNAs (rows) are ordered by hierarchical clustering. The annotation bar indicates the stage (adult vs. fetal).

Additionally, Figure 3 provides a heatmap showing the expressions of the top 20 upregulated circRNAs in both adult and fetal salivary glands across samples.

### 2.3. Functional Analysis

**Figure 4:**
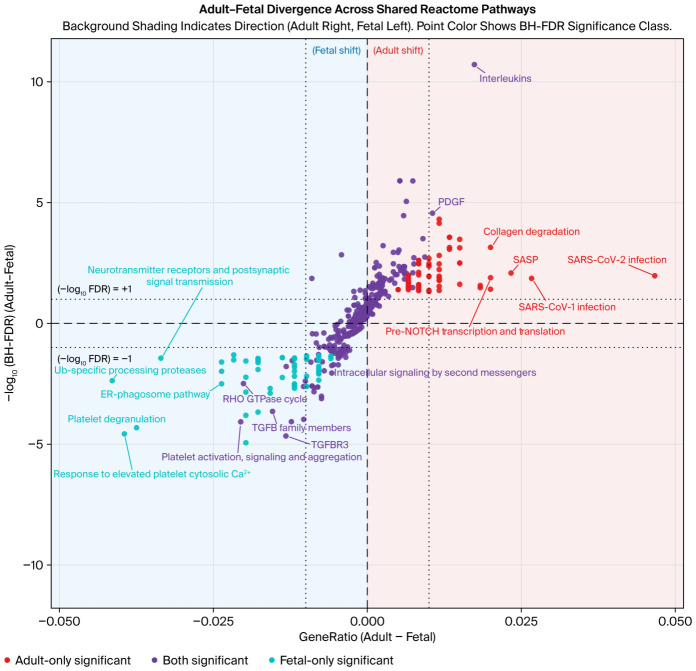
Adult vs. fetal divergence across shared Reactome biological processes. Each point represents a Reactome pathway identified in the enrichment results for the upregulated-circRNA-derived target gene sets in adult and fetal samples. The *x*-axis shows the difference in the GeneRatios between developmental stages (adult GeneRatio minus fetal GeneRatio), where positive values indicate an adult shift and negative values indicate a fetal shift. The *y*-axis shows the difference in enrichment significance between developmental stages, which is calculated as −log10(BH-FDR) in adult salivary glands minus −log10(BH-FDR) in fetal ones; thus, points above zero are more significant in adult glands, and points below zero are more significant in fetal ones. The background shading highlights the direction of the shift (adult—red; fetal—blue). The point colors indicate the FDR significance class for each pathway: red—significant in adult glands only; blue—significant in fetal glands only; purple—significant in both.

To explore the potential functions of the stage-associated circRNAs, the adult- and fetal-upregulated circRNAs were linked to predicted miRNAs using circAtlas 3.0. Only circRNA–miRNA pairs supported by at least two prediction tools (miRanda, PITA, and TargetScan) were retained, and the analysis focused on miRNAs connected to more than one circRNA within each group. These miRNAs were then matched to experimentally supported target genes using miRTarBase (functional MTIs only). The resulting adult- and fetal-specific target gene lists were used for Reactome enrichment to compare the biological pathways associated with each stage (Figure 4).

Table 3 and Table 4 summarize the top 10 Reactome pathways uniquely significant in the fetal and adult target gene sets, respectively, and Table S3 reports the top 10 pathways significant in both groups. Table S4, available as an Excel file, includes the full list of significant Reactome pathways (FDR < 0.05) across the fetal-only, adult-only, and shared results to facilitate filtering, verification, and reuse.

**Figure 5:**
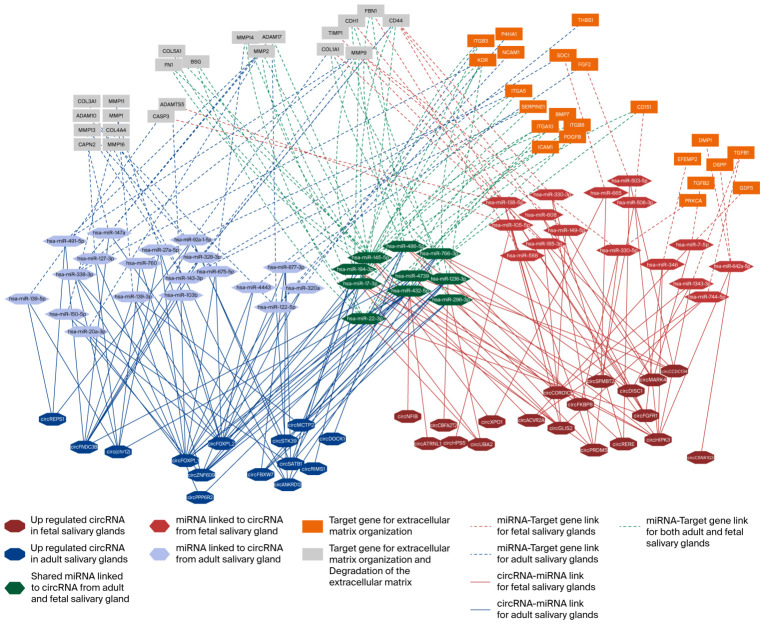
The circRNA–miRNA–target gene network for the extracellular matrix organization and degradation of the extracellular matrix pathways in adult and fetal salivary glands. The gene nodes are grouped according to their related pathways, and the node colors indicate their related tissues, whether from fetal or adult salivary glands or shared between both. miRNAs are shown as the intermediate link between genes and circRNAs, while circRNAs are displayed according to their upregulation in the fetal (red) or adult (blue) salivary glands.

Additionally, a circRNA–miRNA–target gene network was created for the extracellular matrix organization and degradation of the extracellular matrix pathways from the Reactome results (Figure 5).

The network analysis of the extracellular matrix organization and degradation of the extracellular matrix pathways shows substantial overlap in the target genes between adult and fetal salivary glands, with distinct adult- and fetal-specific extensions. The extracellular matrix organization has more genetic influences from fetal salivary gland circRNAs. For the degradation of the extracellular matrix, none of the target genes is unique to this pathway; they all overlap with the extracellular matrix organization, which explains the overlap between the two pathways in the network.

**Figure 6:**
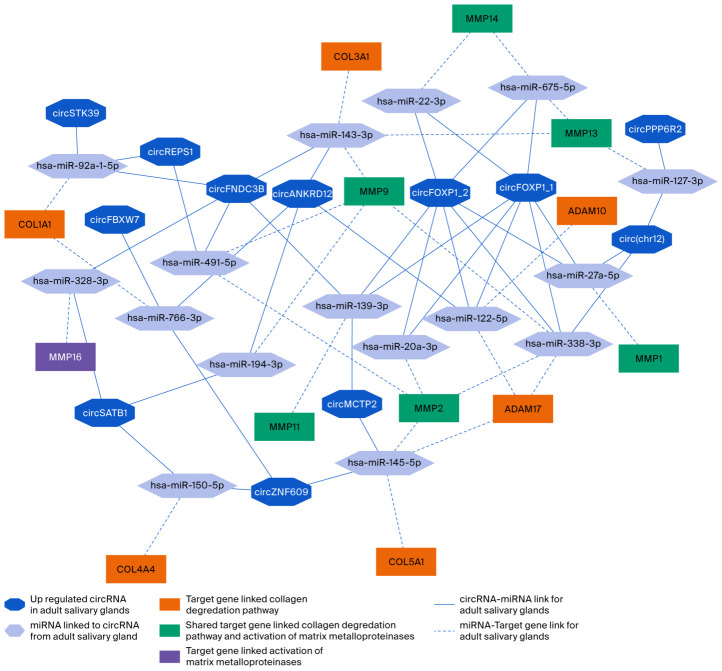
The circRNA–miRNA–target gene network for the collagen degradation and activation of matrix metalloproteinase in adult salivary glands. The octagons represent upregulated circRNAs in adult salivary glands, the hexagons represent miRNAs associated with circRNAs, the orange rectangles represent genes linked only to collagen degradation, the purple rectangles represent genes linked only to matrix metalloproteinase activation, and the green rectangles represent genes shared between both pathways. The solid blue lines indicate circRNA–miRNA linkage, whereas the dashed blue lines indicate miRNA–gene linkage. The network highlights the interconnected adult regulatory module linking circRNAs and miRNAs to collagen-remodeling and metalloproteinase-related target genes.

The collagen degradation and activation of matrix metalloproteinase were adult-specific pathways in the Reactome analysis. The circRNA–miRNA–target gene network (Figure 6) shows a tightly connected matrix-remodeling module centered on six genes that link both pathways: *MMP1*, *MMP2*, *MMP9*, *MMP11*, *MMP13*, and *MMP14*. In addition, several genes were specific to collagen degradation, including *ADAM10*, *ADAM17*, *COL1A1*, *COL3A1*, *COL4A4*, and *COL5A1*, while *MMP16* was uniquely associated with matrix metalloproteinase activation.

These target genes were linked to multiple miRNAs that were associated with the upregulated circRNAs in adult salivary glands.

**Figure 7:**
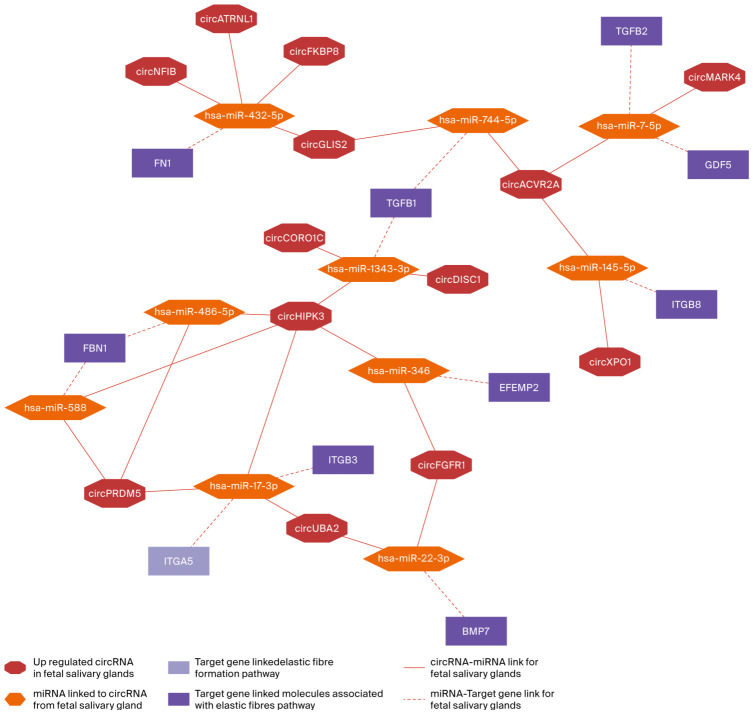
The circRNA–miRNA–target gene network for elastic fiber formation and molecules associated with the elastic fiber formation pathway in fetal salivary glands. The octagons represent fetal-upregulated circRNAs; the hexagons represent miRNAs associated with circRNA; the light purple rectangles represent genes linked only to elastic fiber formation; the dark purple rectangles represent genes linked to both elastic fiber formation and molecules associated with elastic fiber pathways. The solid red lines indicate circRNA–miRNA linkage, whereas the dashed red lines indicate miRNA–gene linkage.

Elastic fiber formation and molecules associated with elastic fiber pathways were fetal-specific in the Reactome analysis. Both pathways shared nine out of ten target genes based on the study analysis, and one gene was unique to elastic fiber formation (Figure 7).

The shared genes included *FN1*, *FBN1*, *EFEMP2*, *ITGA5*, *ITGB3*, *ITGB8*, *BMP7*, *TGFB1*, *TGFB2*, and *GDF5*, indicating that these two fetal pathways are closely interconnected at the gene level. These targets were linked to a relatively small set of miRNAs and upregulated circRNAs from the fetal salivary glands, forming a compact circRNA–miRNA–gene network consistent with the coordinated regulation of elastic fiber and matrix-associated developmental processes in fetal salivary glands.

## 3. Discussion

We characterized the circRNA expression in human salivary glands across different developmental stages using public total RNA-sequencing data from GEO (GSE143702), focusing on adult vs. fetal salivary gland comparisons. A consistent circRNA analysis workflow was created, and only circRNAs supported by at least two back-splicing junction reads per sample were retained for downstream analysis. PCA revealed a distinct separation between adult and fetal samples. This divergence was confirmed via PERMANOVA (R^2^ = 0.118; *p* = 0.001), indicating that the developmental period accounts for a significant portion of the global circRNA variation within salivary gland tissues.

Differential expression analysis identified a set of stage-associated circRNAs, and differential analysis identified eighteen significant (*p* < 0.05) and three suggestive (0.05 < *p* < 0.10) upregulated circRNAs in adult tissues. Fetal samples showed a similar distribution, with eighteen significant and eight suggestive upregulated circRNAs. The stage-associated circRNAs formed clear expression patterns across the samples, as supported by the heatmap of the top adult- and fetal-upregulated circRNAs. These results reveal underlying changes in the tissue circRNA expression landscape and gene-regulatory programs between fetal and adult salivary glands.

Among the top fetal-upregulated circRNAs in salivary gland tissues, circACVR2A(2,3,4).1 was found to be significantly overexpressed compared to its adult counterparts. circACVR2A(2,3,4).1 is derived from the *ACVR2A* gene, which is a member of the transforming growth factor-beta (TGF-β) superfamily [47]. circACVR2A(2,3,4).1 is highly expressed in blood, spinal cord, and retina, as documented in circAtlas 3.0 [48]. While the exact function of the circACVR2A(2,3,4).1 isoform is still subtle, and it is shown to play a context-dependent role in different types of cancer as a tumor suppressor [47]. In bladder cancer, circACVR2A was downregulated and correlated with aggressive characteristics [49]. In addition to the tumor suppressor role, it is speculated to act as an oncogene in hepatocellular carcinoma, leading to cancer progression [47]. Similarly, among the top adult-upregulated circRNAs, circZNF609(2).1 and circDOCK1(2,3,4,5,6,25,26,27).1 were identified as statistically significant. circZNF609(2).1 circRNA plays a role in muscle development and has different functions in different types of cancer [50,51]. Wang et al. suggested that circDOCK1 might be a potential biomarker and treatment approach for Oral Squamous Cell Carcinoma [52]. Despite these findings, none of these circRNAs have been investigated in relation to salivary gland development. This highlights the important knowledge gap and supports the need for functional studies across different contexts in fetal and adult healthy and diseased conditions.

We implemented an integrative circRNA–miRNA–target gene strategy to move beyond differential expression and provide functional context. Stage-enriched circRNAs were linked to predicted miRNAs from the circAtlas 3.0 database [48], and they were then connected to experimentally supported target genes using the miRTarBase database [53] while restricting the scope to functional miRNA target interactions. Additionally, several filters were applied to improve confidence and reduce spurious links. CircRNA–miRNA pairs had to be predicted by at least two miRNA target prediction tools, and miRNAs preferably had to be connected to multiple circRNAs within the same stage sample set. The focus of this strategy was on recurring miRNA signatures across more than two transcripts, providing a more effective structure for viewing bulk interpretation data than that for single-prediction data. Importantly, some circRNAs enriched in adults lacked miRNA associations; therefore, this finding highlights gaps in the available genomic databases. The absence of these links could also suggest that some circRNAs may function in other ways, such as by sequestering proteins or regulating host genes.

In the Reactome divergence analysis, adult and fetal target gene enrichments showed a stage-associated shift in the pathway pattern, consistent with the stage-specific circRNA expression differences observed in the heatmap. Although several pathways are shared between the two developmental stages, their enrichment strengths and gene representations tend to shift toward either the adult or fetal developmental stage, indicating a developmental shift in the pathway rather than a completely distinct pathway.

In addition to the shared pathways, several pathways were uniquely significant in one developmental group. The most significant pathways for fetus-derived targets included G-protein beta: gamma signaling, platelet-related calcium response–degranulation, elastic fiber formation, Rho family GTPase cycles, and mTOR signaling, and the most prominent pathways among the adult-derived targets were those involving WNT-based signaling, NLRP3 inflammasome-associated signaling, collagen breakdown, TP53 activity, and transcriptional program-regulating signaling. The enrichment data gives a concise account of the tissue development. The fetal targets include signaling and matrix organizational processes required in morphogenesis, and the adult targets regulate the homeostasis and maintenance of adult tissue.

However, the enrichment results should be interpreted as pathway-level associations with miRNA target gene sets, rather than a direct indication of pathway activity in salivary gland tissues. For example, Larsen and colleagues found that blocking the PI3K/AKT pathway results in the suppression of cleft formation and the further branching of E13 submandibular gland cultures [54].

A later study by Matsumoto et al. found that WNT signaling suppresses end bud differentiation by inhibiting the AKT pathway [55]. Rho GTPases have been documented to play roles in signal transduction pathways during acinus formation and regeneration, as well as in maintaining epithelial shape, apical polarity, and lumen size [56,57,58,59]. Inhibition of the mTOR signaling pathway by rapamycin reduced the number of branching buds in an ex vivo organ culture, as reported by Sakai and colleagues [60]. Hall, Bradford E et al. showed that the conditional overexpression of the TGF-β1 pathway decreased branching, increased mesenchyme in pups, and caused fibrosis and acinar atrophy in adulthood [61]. In contrast, studies that have investigated WNT signaling in adult salivary glands have found that WIF1 (the Wnt inhibitor) is downregulated while WNT1 (the Wnt activator) is upregulated in human salivary gland tumors [62]. Subsequent investigations by Hai and colleagues using a murine model found that activating Wnt/β-catenin signaling transiently during radiation protects salivary gland function from impairment [63]. A recent study demonstrated that NLRP3 promoted regeneration of the submandibular gland in a rat model [64]. The epigenetic regulators and histone-modifying factors have been studied in the context of salivary gland tumors [65,66]. For example, a previous study demonstrated that HDAC8 is significantly upregulated in adenoid cystic carcinoma [65].

We identified fetal-specific pathways related to the development of elastic fibers and their molecules using the Reactome enrichment tool. This finding aligns with quantitative biophysical experiments on adult and fetal murine submandibular glands, which indicate higher elasticity in the embryonic gland [67]. The involvement of TGFβ1 in salivary gland differentiation and morphogenesis is well documented [68,69]. For example, Peters et al. demonstrated the role of integrin β1 as a key mechanosensor in mechanosensitive mouse salivary gland organ explants cultured on different surface stiffnesses [69], and they also showed that adding exogenous TGFβ1 to explants with inhibited morphogenesis due to abnormal stiffness activates integrin β1 without affecting its expression [69]. Similarly, we identified adult-specific pathways related to collagen degradation and activation of matrix metalloproteinase via the Reactome enrichment tool. Previous studies have reported that MMP-2 and MMP-9 are expressed in the mature ductal cells of healthy adult salivary glands [70]. Moreover, dysregulation of MMP in salivary gland tissue is linked to aging [71], radiation damage [72], and Sjögren’s syndrome (SS) [73], contributing to gland degeneration [70]. circRNAs have gained attention as a potential approach to alleviate fibrosis in various organs, including the heart, liver, lung, kidney, and skin [74], and in addition to their popularity as biomarkers for many conditions, they are promising therapeutic options [75]. Recently, the first intravenous circRNA-based protein replacement therapy for liver fibrosis was investigated in a mouse model [76]. The results showed a notable mitigation in liver fibrosis with the upregulation of MMP as an antifibrotic gene [76]. These findings together provide insights into the development of new therapeutic strategies for salivary gland remodeling and regeneration, particularly for progressive and irreversible progress.

There are several limitations to consider in this study. First, we obtained total RNA-seq data without circRNA enrichment library preparation, thereby reducing the detection sensitivity to low-abundance circRNAs. Second, the analysis was performed on adult vs. fetal salivary glands without stratification by type (parotid, sublingual, or submandibular); thus, these glands were not analyzed separately due to the limited number of samples per group, which reduced the statistical power for circRNA analysis. Third, suitable publicly available human salivary gland tissue cohorts, particularly fetal samples, are limited; therefore, this study was based on a single bulk total RNA-seq dataset, which limits its generalization.

Additionally, the circRNA–miRNA–target gene network should be interpreted as hypothesis-generating evidence rather than as direct proof. The miRNA–target gene component of this functional analysis network was obtained from the miRTarBase database, limited to interactions with experimental validation, and classified as having a strong functional association, which strengthens this part of the analysis. However, the circRNA–miRNA part of the network was obtained from circAtlas 3.0 in silico prediction. Therefore, the functional analysis required further experimental validation.

Lastly, the salivary gland samples are bulk tissues and contain mixed cell populations; therefore, the observed differences in the analysis reflect cellular composition changes rather than cell-specific intrinsic regulation.

Nevertheless, this study offers a structured, stage-oriented circRNA profile of human salivary glands and a narrowed-down set of adult- and fetus-related circRNAs, identified using multivariate separation and expression differences. The integrative miRNA target gene strategy and enrichment divergence framework provide a repeatable format for creating testable proposals for development-related regulatory plans. Future research must confirm top-stage-related circRNAs using an orthogonal system, such as RNase R-treated quantitative assays. Moreover, cell-type factors can be assessed using deconvolution or single-cell methods. Lastly, experimental models should be employed to test candidate circRNA–miRNA–gene axes to define both directionality and mechanisms.

## 4. Materials and Methods

### 4.1. Data Acquisition and Quality Control

Total RNA-seq data and sample annotations were retrieved from the publicly available GEO database (GSE143702) [34]. The RNA-seq data includes two developmental groups: adult salivary glands (n = 13) and fetal salivary glands (n = 14) [34]. In brief, the adult salivary gland samples consist of four parotid glands, six submandibular glands, and three sublingual glands. The parotid and submandibular glands have a balanced sex representation, 50% males and 50% females, while all samples for the sublingual glands were obtained from females [34]. Sublingual gland tissues were obtained from patients with salivary duct stones, with healthy regions identified and separated from the inflamed tissue [34]. Submandibular and parotid gland tissues were collected during surgeries from individuals with head and neck cancer, which was limited to patients who did not receive radiotherapy, chemotherapy, or immunotherapy [34].

Fetal gland samples were obtained from post-mortem fetuses from elective legal abortions, between 22 and 24 weeks of gestation, with written informed consent and an approved institutional review board [34], and they consist of three parotid glands obtained from one female and two males, three submandibular glands obtained from one female and four males, and six sublingual glands obtained from two females and four males [34]. Sex for the fetal samples was confirmed via the expression of the male-specific genes, *UTY* and *KDM5D*.

After the FASTQ files were downloaded, raw RNA-seq reads were assessed for quality using FastQC (v0.11.9). Adapter sequences and low-quality bases were removed with Trimmomatic (v0.39) prior to downstream analyses. Across all samples, the quality metrics were strong, with median Phred scores >30, a balanced nucleotide composition, and no detectable adapter contamination. 

### 4.2. CircRNA Detection and Annotation

The human reference genome (GRCh38) was used to map reads with STAR (v2.7.11b), configured for chimeric junction discovery. Putative circRNAs were then extracted and annotated with CIRCexplorer2 (v2.3.8), using Gencode v39-derived refFlat annotations to assign genomic context.

### 4.3. Quantification and Differential Analysis 

CircRNA candidates supported by ≥2 back-splice junction reads were retained. Differential expressions were assessed with DESeq2 in RStudio (v4.4.2) and were used to model circRNA counts using a negative binomial generalized linear model, with developmental stage as the primary factor. The adult vs. fetal contrast was tested using the Wald test, and *p*-values were adjusted for multiple testing using the Benjamini–Hochberg method. CircRNAs were classified as significant when the adjusted *p*-value was less than 0.05 and as suggestive when the adjusted *p*-value was between 0.05 and 0.10. Tidyverse (v2.0.0) was used for data wrangling, and figures were generated with ggplot2 (v3.5.2). Non-parametric tests were employed for individual comparisons, and group differences were evaluated via PERMANOVA. 

### 4.4. Construction of circRNA-Derived Target Gene Sets

These gene sets were constructed by integrating circRNA–miRNA binding predictions from circAtlas 3.0 [48] with experimentally supported miRNA–target interactions from the miRTarbase database [53].

The top (i.e., most significant and suggestive) circRNAs from both adult and fetal salivary glands were linked to miRNAs using circAtlas 3.0 database prediction. To increase confidence and reduce false links, circRNA–miRNA pairs were retained only when supported by at least two independent prediction tools (miRanda, PITA, and TargetScan).

This approach was used because experimentally validated circRNA–miRNA interaction databases are unavailable, making circAtlas 3.0 a practical resource for in silico analyses of circRNA–miRNA interactions.

For adult salivary glands, the following circRNAs did not have predicted miRNA data in circAtlas 3.0: chr3:114350273-114351878, hsa-ATXN1_0001, chr2:88782733-88792494, and circSETBP1(4).1.

Prioritized higher-confidence regulatory signals were obtained by restricting the scope to miRNAs that interacted with at least two circRNAs within the same group. These filtered miRNAs were mapped to experimentally supported target genes using the miRTarBase database [53], retaining functional miRNA–target interaction (MTI) entries only, thereby generating group-specific circRNA–miRNA–gene linkage tables and corresponding target gene sets for downstream Reactome enrichment analyses. The complete list of circRNA–miRNA–target genes is available in Table S5.

### 4.5. Pathway Enrichment Analysis 

Pathway enrichment analysis was performed to identify the biological processes and pathways most represented in the adult and fetal salivary gland target gene sets. The two gene lists were analyzed separately, and over-represented terms were tested using Reactome pathway databases. *p*-values were adjusted for multiple testing using the Benjamini–Hochberg false-discovery rate (BH-FDR), and terms with adjusted *p*-values < 0.05 were considered significant. Enrichment results were reported as GeneRatios (the fraction of input genes linked to each term), the number of mapped genes, and adjusted *p*-values and were visualized using a consistent dot-plot format to enable direct adult–fetal comparison.

Additionally, a circRNA–miRNA–target gene network was constructed for the extracellular matrix organization and degradation using genes identified from the adult and fetal Reactome enrichment results. Genes were classified as shared, adult-only, or fetal-only and linked to their corresponding miRNAs and upstream circRNAs using the previously derived circRNA–miRNA and miRNA–gene interaction tables, and the resulting network was visualized in Cytoscape 3.10.4 as a single integrated network.

## 5. Conclusions

To the best of our knowledge, this study is the first to provide a circRNA-focused profile of human salivary gland tissues across development stages by comparing fetal and adult salivary glands using a publicly available total RNA-seq cohort. There was a clear separation in the circRNA expression between fetal and adult salivary glands. Network-based functional analysis linking these circRNAs to miRNA–target gene interactions highlights developmental differences in pathway emphasis, including signaling and extracellular matrix remodeling processes. There is growing evidence that circRNAs play crucial regulatory roles in human tissues, including salivary glands. In our study, developmental stage differences in circRNA expression were accompanied by shifts in molecular gene programs. Enrichment results highlighted core developmental signaling modules, including PI3K, AKT, and mTOR, together with WNT, NOTCH, and TGFβ-related pathways, with leading genes such as *AKT1*, *AKT2*, *PTEN*, *PDPK1*, and *MTOR* supporting stage-related differences in growth and differentiation signaling. In parallel, extracellular matrix organization and remodeling pathways were prominent, consistent with developmental matrix assembly and later tissue maturation and maintenance. These findings suggest that circRNAs could serve as molecular targets for understanding and manipulating salivary gland dysfunctions, such as dry-mouth disease, and as a novel therapy for inducing gland regeneration. Although the functional results are hypothesis-generating and based in part on in silico circRNA–miRNA predictions, the outputs provide a useful resource of prioritized circRNAs, miRNAs, and pathways for follow-up validation. Future work should confirm key candidates using orthogonal circRNA assays and evaluate cell type-specific regulation using higher-resolution datasets.

## Figures and Tables

**Table 1:** Top upregulated circRNAs in adult salivary gland tissues.

circAtlas ID	Uniform ID	baseMean	Log2 Fold Change	lfcSE	Stat	*p*-Value	*p*-Adj	Significance
hsa-RIMS1_0021	circRIMS1(L27,28,29).1	5.243	−4.369	0.566	−7.725	<0.0001	<0.0001	Significant
hsa-FBXW7_0005	circFBXW7(3,4).1	8.151	−1.595	0.271	−5.883	<0.0001	<0.0001	Significant
chr3:114350273|114351878	chr3:114350273|114351878	3.071	−3.155	0.612	−5.156	<0.0001	<0.0001	Significant
hsa-FOXP1_0001	circFOXP1(8,9,10,11).1	1.628	−4.026	0.789	−5.106	<0.0001	<0.0001	Significant
hsa-RMST_0004	circ(chr12).49	4.136	−2.477	0.507	−4.885	<0.0001	<0.0001	Significant
hsa-MTUS1_0016	circMTUS1(3L).1	1.583	−3.942	0.808	−4.879	<0.0001	<0.0001	Significant
hsa-FNDC3B_0004	circFNDC3B(5,6).1	11.222	−1.017	0.219	−4.648	<0.0001	0.0001	Significant
hsa-DOCK1_0035	circDOCK1(2,3,4,5,6,25,26,27).1	2.723	−2.708	0.654	−4.142	<0.0001	0.0009	Significant
hsa-MCTP2_0001	circMCTP2(9S,10,11,12,13,14,15,16,L17).1	1.873	−2.860	0.791	−3.614	0.0003	0.0063	Significant
hsa-ZNF609_0001	circZNF609(2).1	3.122	−1.467	0.451	−3.251	0.0012	0.0147	Significant
hsa-SETBP1_0001	circSETBP1(4).1	1.570	−2.675	0.806	−3.320	0.0009	0.0147	Significant
hsa-DOPEY2_0008	circDOP1B(20,21).1	2.221	−2.203	0.678	−3.251	0.0011	0.0147	Significant
hsa-SATB1_0015	circSATB1(2,3,4,5,6,7,8,9).1	1.456	−2.721	0.830	−3.277	0.0011	0.0147	Significant
hsa-ATXN1_0001	circENSG00000288708(7).1	1.864	−2.734	0.838	−3.262	0.0011	0.0147	Significant
chr2:88782733|88792494	chr2:88782733|88792494	0.952	−3.168	1.066	−2.971	0.0030	0.0301	Significant
hsa-STK39_0002	circSTK39(2,3,L4,5).1	1.642	−1.954	0.684	−2.859	0.0043	0.0404	Significant
hsa-REPS1_0015	circREPS1(L5,6,7).1	2.937	−1.482	0.523	−2.835	0.0046	0.0411	Significant
hsa-FOXP1_0003	circFOXP1(8,9,10,11,12).1	1.057	−2.861	1.023	−2.797	0.0052	0.0422	Significant
hsa-RNF13_0008	circRNF13(2,3,4,5,6,7,8).1	4.182	−1.102	0.407	−2.707	0.0068	0.0528	Suggestive
hsa-ANKRD12_0037	circANKRD12(2,3,4,5S,6,7,8).1	4.866	−1.029	0.387	−2.660	0.0078	0.0589	Suggestive
hsa-PPP6R2_0011	circPPP6R2(2,3).1	0.989	−2.789	1.153	−2.418	0.0156	0.0975	Suggestive

**Table 2:** Top upregulated circRNAs in fetal salivary gland tissues.

circAltas ID	Uniform ID	baseMean	log2 Fold Change	lfcSE	Stat	*p*-Value	*p*-Adj	Significance
hsa-SFMBT2_0001	circSFMBT2(5,6,7,8,9).1	1.546	3.854	0.832	4.632	< 0.0001	0.0001	Significant
hsa-FKBP8_0005	circFKBP8(3,4).1	1.834	3.322	0.787	4.220	< 0.0001	0.0008	Significant
hsa-XPO1_0001	circXPO1(2,3,4).1	4.009	1.899	0.457	4.155	< 0.0001	0.0009	Significant
hsa-GLIS2_0002	circGLIS2(2,3).1	1.904	2.956	0.783	3.774	0.0002	0.0039	Significant
hsa-CBFA2T2_0027	circCBFA2T2(4,5).1	2.640	2.014	0.541	3.721	0.0002	0.0045	Significant
hsa-HPS5_0012	circHPS5(15,16).1	2.912	1.761	0.542	3.253	0.0011	0.0147	Significant
hsa-MARK4_0007	circMARK4(RI,3,4).1	1.801	2.610	0.801	3.258	0.0011	0.0147	Significant
hsa-ACVR2A_0001	circACVR2A(2,3,4).1	4.161	1.426	0.427	3.344	0.0008	0.0147	Significant
hsa-DPY30_0005	circMEMO1(6L).3	1.312	3.169	0.958	3.308	0.0009	0.0147	Significant
hsa-ATRNL1_0001	circATRNL1(2,3,L4,5).1	1.145	3.258	1.038	3.140	0.0017	0.0207	Significant
hsa-UBA2_0007	circUBA2(2,3,4,5).1	1.213	3.407	1.116	3.052	0.0023	0.0265	Significant
hsa-STK3_0001	circSTK3(5,6).1	1.524	2.539	0.834	3.043	0.0023	0.0265	Significant
hsa-FGFR1_0001	circFGFR1(3).1	1.676	2.224	0.749	2.971	0.0030	0.0301	Significant
hsa-HIPK3_0001	circHIPK3(2).1	17.715	0.451	0.152	2.973	0.0030	0.0301	Significant
hsa-NFIB_0001	circNFIB(3,4,5,6).1	2.764	1.674	0.571	2.930	0.0034	0.0332	Significant
hsa-LPAR1_0003	circLPAR1(3,4).1	3.408	1.460	0.517	2.823	0.0048	0.0411	Significant
hsa-CSNK1G3_0001	circCSNK1G3(2,3,4).1	5.227	0.943	0.333	2.829	0.0047	0.0411	Significant
hsa-CORO1C_0003	circCORO1C(L7,8).1	3.573	1.343	0.479	2.804	0.0050	0.0422	Significant
hsa-PRDM5_0001	circPRDM5(8,9,10,11,12,13,14).1	1.336	2.306	0.853	2.705	0.0068	0.0528	Suggestive
hsa-CRKL_0001	circCRKL(2).1	1.544	2.146	0.822	2.610	0.0091	0.0666	Suggestive
hsa-MBOAT2_0003	circMBOAT2(2,3,4).1	0.804	3.352	1.307	2.564	0.0104	0.0742	Suggestive
hsa-CCDC134_0001	circCCDC134(2,3,4).1	1.032	2.792	1.116	2.501	0.0124	0.0868	Suggestive
hsa-DISC1_0001	circDISC1(7,8,9).1	1.745	1.881	0.755	2.491	0.0127	0.0871	Suggestive
hsa-RERE_0007	circRERE(5,6,7).1	1.431	2.251	0.927	2.428	0.0152	0.0970	Suggestive
hsa-SPAG16_0008	circSPAG16(4,5,6,7,8,9).1	1.002	2.874	1.177	2.442	0.0146	0.0970	Suggestive
hsa-ASXL1_0001	circASXL1(2,L3,4).1	0.974	2.637	1.084	2.433	0.0150	0.0970	Suggestive

**Table 3:** Top 10 Reactome pathways significant only in fetal salivary gland-derived target gene set (FDR < 0.05).

ID	Description	GeneRatio	Count	Adj *p*-Value	Leading Genes
R-HSA-397795	G-protein beta:gamma signaling	10/507	10	<0.0001	*AKT1*, *AKT2*, *BTK*, *CDC42*, *GNG7*, *PAK1*, *PDPK1*, *PIK3CG*, *PLCB1*, *RHOA*
R-HSA-76005	Response to elevated platelet cytosolic Ca^2+^ levels	20/507	20	<0.0001	*CD36*, *ECM1*, *FERMT3*, *FN1*, *HGF*, *IGF1*, *IGF2*, *ITGB3*, *KNG1*, *PDGFB*, *PRKCA*, *SERPINE1*, *SOD1*, *TGFB1*, *TGFB2*, *TIMP1*, *TIMP3*, *VCL*, *VEGFA*, *VEGFB*
R-HSA-114608	Platelet degranulation	19/507	19	<0.0001	*CD36*, *ECM1*, *FERMT3*, *FN1*, *HGF*, *IGF1*, *IGF2*, *ITGB3*, *KNG1*, *PDGFB*, *SERPINE1*, *SOD1*, *TGFB1*, *TGFB2*, *TIMP1*, *TIMP3*, *VCL*, *VEGFA*, *VEGFB*
R-HSA-1566948	Elastic fiber formation	10/507	10	0.0002	*BMP7*, *EFEMP2*, *FBN1*, *FN1*, *GDF5*, *ITGA5*, *ITGB3*, *ITGB8*, *TGFB1*, *TGFB2*
R-HSA-2129379	Molecules associated with elastic fibers	9/507	9	0.0002	*BMP7*, *EFEMP2*, *FBN1*, *FN1*, *GDF5*, *ITGB3*, *ITGB8*, *TGFB1*, *TGFB2*
R-HSA-9013407	RHOH GTPase cycle	8/507	8	0.0013	*CAV1*, *CSK*, *OSBPL11*, *PAK1*, *PAK4*, *ROCK1*, *ROCK2*, *TFRC*
R-HSA-9013406	RHOQ GTPase cycle	10/507	10	0.0014	*ARHGAP5*, *CAV1*, *CDC42*, *CFTR*, *DLC1*, *GIT1*, *PAK1*, *PAK4*, *SRGAP2*, *TFRC*
R-HSA-165159	MTOR signaling	8/507	8	0.0020	*AKT1*, *AKT2*, *EEF2K*, *EIF4E*, *EIF4EBP1*, *LAMTOR5*, *MTOR*, *RPS6KB1*
R-HSA-9013422	RHOBTB1 GTPase cycle	6/507	6	0.0021	*HNRNPC*, *MYO6*, *ROCK1*, *ROCK2*, *SPEN*, *VIM*
R-HSA-156711	Polo-like-kinase-mediated events	5/507	5	0.0025	*CCNB1*, *CDC25A*, *FOXM1*, *PLK1*, *WEE1*

**Table 4:** Top 10 Reactome pathways significant only in adult-derived target gene set (FDR < 0.05).

ID	Description	GeneRatio	Count	Adj *p*-Value	Leading Genes
R-HSA-3772470	Negative regulation of TCF-dependent signaling by WNT ligand antagonists	7/600	7	<0.0001	*DKK1*, *LRP5*, *SFRP1*, *SFRP2*, *WNT3A*, *WNT4*, *WNT5A*
R-HSA-844456	NLRP3 inflammasome	7/600	7	<0.0001	*CASP1*, *HMOX1*, *NFKB1*, *NLRP3*, *P2RX7*, *RELA*, *TXNIP*
R-HSA-9660826	Purinergic signaling in leishmaniasis infection	8/600	8	0.0003	*CASP1*, *GSDMD*, *HMOX1*, *NFKB1*, *NLRP3*, *P2RX7*, *RELA*, *TXNIP*
R-HSA-9664424	Cell recruitment (pro-inflammatory response)	8/600	8	0.0003	*CASP1*, *GSDMD*, *HMOX1*, *NFKB1*, *NLRP3*, *P2RX7*, *RELA*, *TXNIP*
R-HSA-4791275	Signaling by WNT in cancer	9/600	9	0.0003	*APC*, *CTNNB1*, *DKK1*, *FZD4*, *FZD8*, *GSK3B*, *LRP5*, *PPP2CA*, *WNT3A*
R-HSA-1442490	Collagen degradation	12/600	12	0.0007	*ADAM10*, *ADAM17*, *COL1A1*, *COL3A1*, *COL4A4*, *COL5A1*, *MMP1*, *MMP11*, *MMP13*, *MMP14*, *MMP2*, *MMP9*
R-HSA-6804758	Regulation of TP53 activity through acetylation	8/600	8	0.0007	*AKT1*, *AKT2*, *AKT3*, *EP300*, *HDAC1*, *HDAC2*, *ING5*, *TP53*
R-HSA-8853884	Transcriptional regulation by VENTX	9/600	9	0.0007	*AGO3*, *CCND1*, *CSF1R*, *CTNNB1*, *IL6*, *NFKB1*, *RELA*, *TP53*, *UBE2C*
R-HSA-4641262	Disassembly of destruction complex and recruitment of AXIN to membrane	8/600	8	0.0009	*APC*, *CTNNB1*, *DVL1*, *GSK3B*, *LRP5*, *PPP2CA*, *WNT1*, *WNT3A*
R-HSA-3238698	WNT ligand biogenesis and trafficking	7/600	7	0.0015	*WNT1*, *WNT10A*, *WNT11*, *WNT2B*, *WNT3A*, *WNT4*, *WNT5A*

## Data Availability

All data analyzed in this study are publicly available from the Gene Expression Omnibus (GEO) under accession number GSE143702. The datasets can be accessed at https://www.ncbi.nlm.nih.gov/geo/ (last accessed on 1 February 2026) using the provided accession numbers.

## Supplementary Materials

The following supporting information can be downloaded at: https://www.mdpi.com/article/10.3390/ijms27083608/s1.

## Abbreviations

The following abbreviations are used in this manuscript:

BH-FDR	Benjamini–Hochberg false-discovery rate
ciRNA	Circular intronic RNA
circRNA(s)	Circular RNA(s)
CIRCexplorer2	Circular RNA parsing and annotation tool
DESeq2	Differential expression analysis package
eiciRNA	Exon–intron circular RNA
ecircRNA	Exonic circular RNA
FASTQ	Sequencing read file format
FGF	Fibroblast growth factor
GEO	Gene Expression Omnibus
GO BP	Gene Ontology Biological Process
GRCh38	Genome Reference Consortium Human Build 38
MTI/MTIs	miRNA–target interaction(s)
miRNA(s)	MicroRNA(s)
miRTarBase	MicroRNA target interaction database
PCA	Principal component analysis
PERMANOVA	Permutational multivariate analysis of variance
RNA-seq	RNA sequencing
scRNA-seq	Single-cell RNA sequencing
STAR	Spliced Transcript Alignment to a Reference
TGFβ	Transforming growth factor beta

## References

[1] Chibly A.M., Aure M.H., Patel V.N., Hoffman M.P. (2022). Salivary gland function, development, and regeneration. Physiol. Rev..

[2] Suzuki A., Ogata K., Iwata J. (2021). Cell signaling regulation in salivary gland development. Cell Mol. Life Sci..

[3] Pedersen A.M.L., Sorensen C.E., Proctor G.B., Carpenter G.H., Ekstrom J. (2018). Salivary secretion in health and disease. J. Oral Rehabil..

[4] Pedersen A., Sorensen C.E., Proctor G.B., Carpenter G.H. (2018). Salivary functions in mastication, taste and textural perception, swallowing and initial digestion. Oral Dis..

[5] Patel V.N., Hoffman M.P. (2014). Salivary gland development: A template for regeneration. Semin. Cell Dev. Biol..

[6] de Paula F., Teshima T.H.N., Hsieh R., Souza M.M., Nico M.M.S., Lourenco S.V. (2017). Overview of Human Salivary Glands: Highlights of Morphology and Developing Processes. Anat. Rec..

[7] Valstar M.H., de Bakker B.S., Steenbakkers R., de Jong K.H., Smit L.A., Klein Nulent T.J.W., van Es R.J.J., Hofland I., de Keizer B., Jasperse B. (2021). The tubarial salivary glands: A potential new organ at risk for radiotherapy. Radiother. Oncol..

[8] Ebrahim A., Reich C., Wilde K., Salim A.M., Hyrcza M.D., Willetts L. (2025). A comprehensive analysis of the tubarial glands. Anat. Rec..

[9] Iwanaga J., Ibaragi S., Nakano K., Takeshita Y., Tubbs R.S. (2021). No convincing evidence for the presence of tubarial salivary glands: A letter to the editor regarding “The tubarial salivary glands: A potential new organ at risk for radiotherapy”. Radiother. Oncol..

[10] Carlson E.R., Ord R.A. (2022). Salivary Gland Pathology: Diagnosis and Management.

[11] Zurek M., Fus L., Niemczyk K., Rzepakowska A. (2023). Salivary gland pathologies: Evolution in classification and association with unique genetic alterations. Eur. Arch. Oto-Rhino-Laryngol..

[12] Kim M.J., Milliren A., Gerold D.J. (2024). Salivary Gland Disorders: Rapid Evidence Review. Am. Fam. Physician.

[13] Bishop J.A., Thompson L.D.R., Wakely P.E., Weinreb I., American Registry of Pathology (2021). Tumors of the Salivary Glands.

[14] Schubert M.M., Izutsu K.T. (1987). Iatrogenic causes of salivary gland dysfunction. J. Dent. Res..

[15] Gunning J.A., Limesand K.H. (2024). Chronic Phenotypes Underlying Radiation-Induced Salivary Gland Dysfunction. J. Dent. Res..

[16] Meijer T.W.H., Scandurra D., Langendijk J.A. (2020). Reduced radiation-induced toxicity by using proton therapy for the treatment of oropharyngeal cancer. Br. J. Radiol..

[17] Plemons J.M., Al-Hashimi I., Marek C.L., American Dental Association Council on Scientific A (2014). Managing xerostomia and salivary gland hypofunction: Executive summary of a report from the American Dental Association Council on Scientific Affairs. J. Am. Dent. Assoc..

[18] Guggenheimer J., Moore P.A. (2003). Xerostomia: Etiology, recognition and treatment. J. Am. Dent. Assoc..

[19] Murphy Dourieu E., Lisiecka D., Evans W., Sheahan P. (2025). Xerostomia: A silent burden for people receiving palliative care—A qualitative descriptive study. BMC Palliat. Care.

[20] Rothova M., Thompson H., Lickert H., Tucker A.S. (2012). Lineage tracing of the endoderm during oral development. Dev. Dyn..

[21] Wang J., Laurie G.W. (2004). Organogenesis of the exocrine gland. Dev. Biol..

[22] Wrynn T., Min S., Horeth E., Osinski J., Sinha S., Romano R.A. (2024). DeltaNp63 regulates Sfrp1 expression to direct salivary gland branching morphogenesis. PLoS ONE.

[23] Musicant A.M., Billington J.M.R., Damrauer J.S., Modliszewski J.L., Landau L.J.B., Tsai Y.H., Mehta J.H., Powers J., Betancourt R., Sekhri R. (2025). An FGFR-p53 developmental signaling axis drives salivary cancer progression. Oncogene.

[24] Iber D., Menshykau D. (2013). The control of branching morphogenesis. Open Biol..

[25] Dang H., Lin A.L., Zhang B., Zhang H.M., Katz M.S., Yeh C.K. (2009). Role for Notch signaling in salivary acinar cell growth and differentiation. Dev. Dyn..

[26] Garcia-Gallastegui P., Ibarretxe G., Garcia-Ramirez J.J., Baladron V., Aurrekoetxea M., Nueda M.L., Naranjo A.I., Santaolalla F., Sanchez-del Rey A., Laborda J. (2014). DLK1 regulates branching morphogenesis and parasympathetic innervation of salivary glands through inhibition of NOTCH signalling. Biol. Cell.

[27] Zinski J., Tajer B., Mullins M.C. (2018). TGF-beta Family Signaling in Early Vertebrate Development. Cold Spring Harb. Perspect. Biol..

[28] Kahata K., Maturi V., Moustakas A. (2018). TGF-beta Family Signaling in Ductal Differentiation and Branching Morphogenesis. Cold Spring Harb. Perspect. Biol..

[29] Jaskoll T., Choy H.A., Melnick M. (1994). Glucocorticoids, TGF-beta, and embryonic mouse salivary gland morphogenesis. J. Craniofac Genet. Dev. Biol..

[30] Lau W.W., Hardt M., Zhang Y.H., Freire M., Ruhl S. (2021). The Human Salivary Proteome Wiki: A Community-Driven Research Platform. J. Dent. Res..

[31] Ehnes D.D., Alghadeer A., Hanson-Drury S., Zhao Y.T., Tilmes G., Mathieu J., Ruohola-Baker H. (2022). Sci-Seq of Human Fetal Salivary Tissue Introduces Human Transcriptional Paradigms and a Novel Cell Population. Front. Dent. Med..

[32] Horeth E., Bard J., Che M., Wrynn T., Song E.A.C., Marzullo B., Burke M.S., Popat S., Loree T., Zemer J. (2023). High-Resolution Transcriptomic Landscape of the Human Submandibular Gland. J. Dent. Res..

[33] Horeth E., Wrynn T., Osinski J.M., Glathar A., Bard J., Burke M.S., Popat S., Loree T., Nagai M., Phillips R. (2025). Multimodal Exploration Offers Novel Insights into the Transcriptomic and Epigenomic Landscape of the Human Submandibular Glands. Cells.

[34] Saitou M., Gaylord E.A., Xu E., May A.J., Neznanova L., Nathan S., Grawe A., Chang J., Ryan W., Ruhl S. (2020). Functional Specialization of Human Salivary Glands and Origins of Proteins Intrinsic to Human Saliva. Cell Rep..

[35] Khanna P., Jenney K., Tai A.K., Maron J.L. (2021). Salivary RNA sequencing highlights a sex-specific developmental time course towards oral feeding maturation in the newborn. Pediatr. Med..

[36] Maron J.L., Hwang J.S., Pathak S., Ruthazer R., Russell R.L., Alterovitz G. (2015). Computational gene expression modeling identifies salivary biomarker analysis that predict oral feeding readiness in the newborn. J. Pediatr..

[37] Maron J.L., Johnson K.L., Dietz J.A., Chen M.L., Bianchi D.W. (2012). Neuropeptide Y2 receptor (NPY2R) expression in saliva predicts feeding immaturity in the premature neonate. PLoS ONE.

[38] Zimmerman E., Maki M., Maron J. (2016). Salivary FOXP2 expression and oral feeding success in premature infants. Mol. Case Stud..

[39] Zimmerman E., Maron J.L. (2016). FOXP2 gene deletion and infant feeding difficulties: A case report. Mol. Case Stud..

[40] Baiddou C., Knidiri M., Ghazi B., Obaid A.A., Hamdi S., Errafii K. (2026). The circular RNA landscape: Biogenesis, functions, identification pipelines, and biomedical applications. Non-Coding RNA Res..

[41] Verduci L., Tarcitano E., Strano S., Yarden Y., Blandino G. (2021). CircRNAs: Role in human diseases and potential use as biomarkers. Cell Death Dis..

[42] Greene J., Baird A.-M., Brady L., Lim M., Gray S.G., McDermott R., Finn S.P. (2017). Circular RNAs: Biogenesis, function and role in human diseases. Front. Mol. Biosci..

[43] Kristensen L.S., Andersen M.S., Stagsted L.V., Ebbesen K.K., Hansen T.B., Kjems J. (2019). The biogenesis, biology and characterization of circular RNAs. Nat. Rev. Genet..

[44] Chen L.-L. (2020). The expanding regulatory mechanisms and cellular functions of circular RNAs. Nat. Rev. Mol. Cell Biol..

[45] Yuan H., Liao X., Hu D., Guan D., Tian M. (2024). Back to the origin: Mechanisms of circRNA-directed regulation of host genes in human disease. Non-Coding RNA.

[46] Wang A., Wang J., Mao M., Zhao X., Li Q., Xuan R., Li F., Chao T. (2023). Analyses of lncRNAs, circRNAs, and the interactions between ncRNAs and mRNAs in goat submandibular glands reveal their potential function in immune regulation. Genes.

[47] Fei D., Wang F., Wang Y., Chen J., Chen S., Fan L., Yang L., Ren Q., Duangmano S., Du F. (2024). Circular RNA ACVR2A promotes the progression of hepatocellular carcinoma through mir-511-5p targeting PI3K-Akt signaling pathway. Mol. Cancer.

[48] Wu W., Zhao F., Zhang J. (2024). circAtlas 3.0: A gateway to 3 million curated vertebrate circular RNAs based on a standardized nomenclature scheme. Nucleic Acids Res..

[49] Dong W., Bi J., Liu H., Yan D., He Q., Zhou Q., Wang Q., Xie R., Su Y., Yang M. (2019). Circular RNA ACVR2A suppresses bladder cancer cells proliferation and metastasis through miR-626/EYA4 axis. Mol. Cancer.

[50] Legnini I., Di Timoteo G., Rossi F., Morlando M., Briganti F., Sthandier O., Fatica A., Santini T., Andronache A., Wade M. (2017). Circ-ZNF609 Is a Circular RNA that Can Be Translated and Functions in Myogenesis. Mol. Cell.

[51] Zhang X., Zhao Y., Kong P., Han M., Li B. (2019). Expression of circZNF609 is Down-Regulated in Colorectal Cancer Tissue and Promotes Apoptosis in Colorectal Cancer Cells by Upregulating p53. Med. Sci. Monit..

[52] Wang L., Wei Y., Yan Y., Wang H., Yang J., Zheng Z., Zha J., Bo P., Tang Y., Guo X. (2018). CircDOCK1 suppresses cell apoptosis via inhibition of miR-196a-5p by targeting BIRC3 in OSCC. Oncol. Rep..

[53] Cui S., Yu S., Huang H.-Y., Lin Y.-C., Huang Y., Zhang B., Xiao J., Zuo H., Wang J., Li Z. (2025). miRTarBase 2025: Updates to the collection of experimentally validated microRNA–target interactions. Nucleic Acids Res..

[54] Larsen M., Hoffman M.P., Sakai T., Neibaur J.C., Mitchell J.M., Yamada K.M. (2003). Role of PI 3-kinase and PIP3 in submandibular gland branching morphogenesis. Dev. Biol..

[55] Matsumoto S., Kurimoto T., Taketo M.M., Fujii S., Kikuchi A. (2016). The WNT/MYB pathway suppresses KIT expression to control the timing of salivary proacinar differentiation and duct formation. Development.

[56] Xu N., Keung B., Myat M.M. (2008). Rho GTPase controls invagination and cohesive migration of the Drosophila salivary gland through Crumbs and Rho-kinase. Dev. Biol..

[57] Nikolaidou K.K., Barrett K. (2004). A Rho GTPase signaling pathway is used reiteratively in epithelial folding and potentially selects the outcome of Rho activation. Curr. Biol..

[58] Shiratsuchi H., Shimizu O., Saito T., Mashimo T., Yonehara Y. (2012). Immunohistological study of small Rho GTPases and beta-catenin during regeneration of the rat submandibular gland. J. Mol. Histol..

[59] Crema V.O., Hamassaki D.E., Santos M.F. (2006). Small Rho GTPases are important for acinus formation in a human salivary gland cell line. Cell Tissue Res..

[60] Sakai M., Fukumoto M., Ikai K., Ono Minagi H., Inagaki S., Kogo M., Sakai T. (2019). Role of the mTOR signalling pathway in salivary gland development. FEBS J..

[61] Hall B.E., Zheng C., Swaim W.D., Cho A., Nagineni C.N., Eckhaus M.A., Flanders K.C., Ambudkar I.S., Baum B.J., Kulkarni A.B. (2010). Conditional overexpression of TGF-beta1 disrupts mouse salivary gland development and function. Lab. Investig..

[62] Queimado L., Obeso D., Hatfield M.D., Yang Y., Thompson D.M., Reis A.M. (2008). Dysregulation of Wnt pathway components in human salivary gland tumors. JAMA Otolaryngol.–Head Neck Surg..

[63] Hai B., Yang Z., Shangguan L., Zhao Y., Boyer A., Liu F. (2012). Concurrent transient activation of Wnt/beta-catenin pathway prevents radiation damage to salivary glands. Int. J. Radiat. Oncol. Biol. Phys..

[64] Zhang Y., Zhang H., Li D., Zhang Y., Qin L. (2025). NLRP3 Mediates Submandibular Gland Regeneration in Duct Ligation/De-Ligation Model. Oral Dis..

[65] Manou M., Loupis T., Vrachnos D.M., Katsoulas N., Theocharis S., Kanakoglou D.S., Basdra E.K., Piperi C., Papavassiliou A.G. (2023). Enhanced Transcriptional Signature and Expression of Histone-Modifying Enzymes in Salivary Gland Tumors. Cells.

[66] Dos Santos E.S., Ramos J.C., Normando A.G.C., Mariano F.V., Paes Leme A.F. (2020). Epigenetic alterations in salivary gland tumors. Oral Dis..

[67] Mosier A.P., Peters S.B., Larsen M., Cady N.C. (2014). Microfluidic platform for the elastic characterization of mouse submandibular glands by atomic force microscopy. Biosensors.

[68] Peters S.B., Naim N., Nelson D.A., Mosier A.P., Cady N.C., Larsen M. (2014). Biocompatible tissue scaffold compliance promotes salivary gland morphogenesis and differentiation. Tissue Eng. Part A.

[69] Peters S.B., Nelson D.A., Kwon H.R., Koslow M., DeSantis K.A., Larsen M. (2015). TGFbeta signaling promotes matrix assembly during mechanosensitive embryonic salivary gland restoration. Matrix Biol..

[70] Marinkovic M., Tran O.N., Wang H., Abdul-Azees P., Dean D.D., Chen X.D., Yeh C.K. (2023). Extracellular matrix turnover in salivary gland disorders and regenerative therapies: Obstacles and opportunities. J. Oral Biol. Craniofac. Res..

[71] Tumer M.K., Cicek M. (2018). Differential immunohistochemical expression of type I collagen and matrix metalloproteinase 2 among major salivary glands of young and geriatric mice. J. Appl. Oral Sci..

[72] Lombaert I.M.A., Patel V.N., Jones C.E., Villier D.C., Canada A.E., Moore M.R., Berenstein E., Zheng C., Goldsmith C.M., Chorini J.A. (2020). CERE-120 Prevents Irradiation-Induced Hypofunction and Restores Immune Homeostasis in Porcine Salivary Glands. Mol. Ther. Methods Clin. Dev..

[73] Perez P., Goicovich E., Alliende C., Aguilera S., Leyton C., Molina C., Pinto R., Romo R., Martinez B., Gonzalez M.J. (2000). Differential expression of matrix metalloproteinases in labial salivary glands of patients with primary Sjogren’s syndrome. Arthritis Rheum..

[74] Wei L., Liu L., Bai M., Ning X., Sun S. (2023). CircRNAs: Versatile players and new targets in organ fibrosis. Cell Commun. Signal.

[75] Ye D., Gong M., Deng Y., Fang S., Cao Y., Xiang Y., Shen Z. (2022). Roles and clinical application of exosomal circRNAs in the diagnosis and treatment of malignant tumors. J. Transl. Med..

[76] Zhong J., Zhang Z., Xiao L., Wang C., Yang Y., Zhang Q., Wang Z. (2026). Circular RNA encoding relaxin-2 as a potential therapy for liver fibrosis. Mol. Ther. Nucleic Acids.

