# Supplementary material for: Developmental Differences in Circular RNA Expression Between Adult and Fetal Human Salivary Glands Based on Public Total RNA-Sequencing Data
Source: Int J Mol Sci. 2026 Apr 18;27(8):3608. doi: 10.3390/ijms27083608 (PMC13116067; doi:10.3390/ijms27083608)
Supplement: Supplementary file 1 [file ijms-27-03608-s001.zip › Table S1 circRNA count per sample by stage and gland type.pdf]

Table S1: circRNA count per sample by stage and gland type

Sample ID	circRNAs count (≥ 2 reads)	Stage	Gland Type
SRR10898041	419	Adult	Sublingual
SRR10898042	563	Adult	Parotid
SRR10898043	376	Adult	Parotid
SRR10898044	491	Adult	Submandibular
SRR10898045	399	Adult	Parotid
SRR10898046	290	Adult	Submandibular
SRR10898047	390	Adult	Parotid
SRR10898048	598	Adult	Sublingual
SRR10898049	764	Adult	Submandibular
SRR10898050	527	Adult	Submandibular
SRR10898051	624	Adult	Submandibular
SRR10898052	500	Adult	Submandibular
SRR10898053	634	Adult	Sublingual
SRR10898054	365	Fetal	Sublingual
SRR10898055	353	Fetal	Parotid
SRR10898056	557	Fetal	Sublingual
SRR10898057	462	Fetal	Submandibular
SRR10898058	303	Fetal	Parotid
SRR10898059	348	Fetal	Submandibular
SRR10898060	158	Fetal	Sublingual
SRR10898061	169	Fetal	Sublingual
SRR10898063	336	Fetal	Submandibular
SRR10898064	681	Fetal	Sublingual
SRR10898065	520	Fetal	Submandibular
SRR10898066	445	Fetal	Sublingual
SRR10898067	393	Fetal	Submandibular
SRR10898068	403	Fetal	Parotid
