# Supplementary material for: Developmental Differences in Circular RNA Expression Between Adult and Fetal Human Salivary Glands Based on Public Total RNA-Sequencing Data
Source: Int J Mol Sci. 2026 Apr 18;27(8):3608. doi: 10.3390/ijms27083608 (PMC13116067; doi:10.3390/ijms27083608)
Supplement: Supplementary file 1 [file ijms-27-03608-s001.zip › Table S2 Summary statistics for circRNAs detected per stage and gland type.pdf]

Table S2: Summary statistics for circRNAs detected per stage and gland type

Stage	Gland type	n	mean circRNAs count (≥ 2 reads)	SD	Min	Max
Adult	Parotid	4	432	87.8	376	563
Adult	Sublingual	3	550.3	115.2	419	634
Adult	Submandibular	6	532.7	157.2	290	764
Fetal	Parotid	3	353	50	303	403
Fetal	Sublingual	6	395.8	209.1	158	681
Fetal	Submandibular	5	411.8	78.1	336	520
Wilcoxon p-value	0.044					
