# Supplementary material for: Developmental Differences in Circular RNA Expression Between Adult and Fetal Human Salivary Glands Based on Public Total RNA-Sequencing Data
Source: Int J Mol Sci. 2026 Apr 18;27(8):3608. doi: 10.3390/ijms27083608 (PMC13116067; doi:10.3390/ijms27083608)
Supplement: Supplementary file 1 [file ijms-27-03608-s001.zip › Table S3 Top 10 Reactome pathways significant in both adult and fetal-derived target gene sets .pdf]

Table S3: Top 10 Reactome pathways significant in both adult and fetal-derived target gene sets

ID	Description	GeneRatio Adult	GeneRatio Fetal	Count Adult	Count Fetal	Delta GeneRatio (Adult - Fetal)	Adj p-value in Adult	Adj p-value in fetal	Direction	leading genes for adult	leading genes for fetal
R-HSA-449147	Signaling by Interleukins	98/600	74/507	98	74	0.017376726	$p < 0.0001$	$p < 0.0001$	Adult shift	*AKT1, ARF1, BCL2, BCL2L1, BCL6, BIRC5, CANX, CASP1, CASP3, CBL, CCL2, CCL5, CCND1, CD36, CD4, CDC42, CDKN1A, CISH, CREB1, CSF1R, CXCL2, DUSP4, FGF2, FN1, FOS, FOXO1, FSCN1, FYN, GRB2, GSDMD, HIF1A, HMGB1, HMOX1, HNRNPA2B1, ICAM1, IL11, IL13RA1, IL15RA, IL23R, IL4, IL6, IRS1, IRS2, JAK1, JAK2, JUN, LGALS9, LIF, MAOA, MAP2K7, MAP3K7, MAP3K8, MAPK1, MAPK11, MAPK14, MAPK7, MCL1, MMP1, MMP2, MMP9, MUC1, MYC, NANOG, NFKB1, NOD2, PDCD4, PIK3CA, PIK3R1, PIK3R2, PIK3R3, PIM1, PPP2CA, PSMA3, PTGS2, PTPN4, RAP1B, RELA, RPLP0, SDC1, SMAD3, SMARCA4, SOCS1, SOCS2, SOD2, SOX2, STAT1, STAT3, STAT5B, STAT6, TNF, TP53, TRAF2, TWIST1, VEGFA, VIM, YES1, YWHAZ, ZEB1*	*AKT1, BCL2, BCL2L1, BCL6, CAPZA1, CASP3, CBL, CCND1, CD36, CDC42, CDKN1A, CREB1, CSF1R, DUSP4, FGF2, FN1, FOS, FOXO1, FOXO3, FSCN1, FYN, GRB2, GSDMD, HGF, HIF1A, HMGB1, ICAM1, IKBKB, IL23R, IL6, IRAK1, IRS1, IRS2, JAK1, LCN2, LGALS9, LIF, MAOA, MAPK1, MAPK14, MCL1, MMP2, MMP9, MUC1, MYC, MYD88, NANOG, PIK3CD, PIK3R1, PIK3R3, PIM1, PSME3, PTGS2, RELA, RPLP0, SDC1, SMAD3, SOCS2, SOCS3, SOD1, SOD2, SOX2, STAT1, STAT3, TGFB1, TIMP1, TNIP2, TRAF2, TWIST1, VEGFA, VIM, YES1, YWHAZ, ZEB1*

R-HSA-5663202	Diseases of signal transduction by growth factor receptors and second messengers	94/600	84/507	94	84	-0.00901381	p < 0.0001	p < 0.0001	Fetal shift	*ADAM10, ADAM17, AGO2, AGO3, AKT1, AKT2, AKT3, APC, ATG7, BCL2L1, BDNF, BRAF, CBL, CD28, CDK8, CDKN1A, CDKN1B, CREB1, CTNNB1, DKK1, DLL1, DNMT1, EGFR, EP300, ERBB2, ERBB3, ESR1, FGF1, FGF10, FGF2, FGF20, FLT3, FN1, FOXM1, FOXO1, FRS2, FYN, FZD4, FZD8, GRB2, GSK3B, HDAC1, HDAC11, HDAC2, HDAC4, HDAC6, HDAC9, HRAS, IRS1, IRS2, ITGB3, JAK2, JUN, KDR, KIT, KITLG, KRAS, LRP5, MAPK1, MCL1, MDM2, MECP2, MET, MYC, NF1, NOTCH1, NRAS, NTRK3, PDGFB, PIK3CA, PIK3R1, PIK3R2, PIK3R3, PIM1, PPP2CA, PSMA3, PTEN, RAC1, RAF1, RAP1B, RB1, SMAD2, SMAD3, SMAD4, SND1, SPTBN1, STAT1, STAT3, STAT5B, TGFBR2, TP53, TWIST1, WNT3A, YES1*	*ACTB, ADAM17, AGO1, AGO2, AKT1, AKT2, ARRB1, ATG7, BCL11A, BCL2L1, BDNF, BRAF, CBL, CCNB1, CD28, CDKN1A, CREB1, CSK, DLL1, DNMT1, EGFR, ERBB2, ERBB3, ESR1, FBXW7, FGF10, FGF2, FGF20, FGF8, FGFR1, FN1, FOXM1, FOXO1, FOXO3, FRS2, FYN, GRB2, GSK3B, HDAC1, HDAC11, HDAC2, HDAC4, HDAC6, HDAC9, HGF, HRAS, IRS1, IRS2, ITGB3, KDR, KRAS, MAP3K11, MAPK1, MCL1, MDM2, MECP2, MET, MTOR, MYC, NOTCH1, NRAS, NTRK3, PDGFB, PDPK1, PIK3CD, PIK3R1, PIK3R3, PIM1, PSME3, PTEN, RAF1, SMAD3, SMAD4, SPTBN1, STAT1, STAT3, TGFB1, TGFBR1, TGFBR2, TNKS2, TWIST1, VCL, WNT3A, YES1*
R-HSA-6785807	Interleukin-4 and Interleukin-13 signaling	46/600	41/507	46	41	-0.00420118	p < 0.0001	p < 0.0001	Fetal shift	*AKT1, BCL2, BCL2L1, BCL6, BIRC5, CCL2, CCND1, CD36, CDKN1A, FGF2, FN1, FOS, FOXO1, FSCN1, HIF1A, HMOX1, ICAM1, IL13RA1, IL23R, IL4, IL6, JAK1, JAK2, LIF, MAOA, MCL1, MMP1, MMP2, MMP9, MUC1, MYC, NANOG, PIK3R1, PIM1, PTGS2, SOCS1, SOX2, STAT1, STAT3, STAT6, TNF, TP53, TWIST1, VEGFA, VIM, ZEB1*	*AKT1, BCL2, BCL2L1, BCL6, CCND1, CD36, CDKN1A, FGF2, FN1, FOS, FOXO1, FOXO3, FSCN1, HGF, HIF1A, ICAM1, IL23R, IL6, JAK1, LCN2, LIF, MAOA, MCL1, MMP2, MMP9, MUC1, MYC, NANOG, PIK3R1, PIM1, PTGS2, SOCS3, SOX2, STAT1, STAT3, TGFB1, TIMP1, TWIST1, VEGFA, VIM, ZEB1*

R-HSA-2219528	PI3K/AKT Signaling in Cancer	36/600	34/507	36	34	-0.00706114	p < 0.0001	p < 0.0001	Fetal shift	*AKT1, AKT2, AKT3, BDNF, CD28, CDKN1A, CDKN1B, CREB1, EGFR, ERBB2, ERBB3, ESR1, FGF1, FGF10, FGF2, FGF20, FLT3, FOXO1, FRS2, FYN, GRB2, GSK3B, IRS1, IRS2, KIT, KITLG, MDM2, MET, NTRK3, PDGFB, PIK3CA, PIK3R1, PIK3R2, PIK3R3, PTEN, RAC1*	*AKT1, AKT2, BDNF, CD28, CDKN1A, CREB1, EGFR, ERBB2, ERBB3, ESR1, FGF10, FGF2, FGF20, FGF8, FGFR1, FOXO1, FOXO3, FRS2, FYN, GRB2, GSK3B, HGF, IRS1, IRS2, MDM2, MET, MTOR, NTRK3, PDGFB, PDPK1, PIK3CD, PIK3R1, PIK3R3, PTEN*
R-HSA-1257604	PIP3 activates AKT signaling	55/600	51/507	55	51	-0.00892505	p < 0.0001	p < 0.0001	Fetal shift	*AGO2, AGO3, AKT1, AKT2, AKT3, BDNF, BMI1, CD28, CDKN1A, CDKN1B, CREB1, CSNK2A1, EGFR, ERBB2, ERBB3, ESR1, EZH2, FGF1, FGF10, FGF2, FGF20, FLT3, FOXO1, FRS2, FYN, GRB2, GSK3B, HDAC1, HDAC2, IRS1, IRS2, JUN, KIT, KITLG, MAPK1, MDM2, MET, MTA1, NTRK3, PDGFB, PHLPP1, PIK3CA, PIK3R1, PIK3R2, PIK3R3, PPP2CA, PREX2, PSMA3, PTEN, RAC1, RCOR1, SNAI1, SNAI2, SUZ12, TP53*	*AGO1, AGO2, AKT1, AKT2, BDNF, CD28, CDKN1A, CREB1, EGFR, ERBB2, ERBB3, ESR1, EZH2, FGF10, FGF2, FGF20, FGF8, FGFR1, FOXO1, FOXO3, FRS2, FYN, GRB2, GSK3B, HDAC1, HDAC2, HGF, IRAK1, IRS1, IRS2, LAMTOR5, MAPK1, MDM2, MET, MTA1, MTOR, MYD88, NTRK3, PDGFB, PDPK1, PIK3CD, PIK3R1, PIK3R3, PSME3, PTEN, RCOR1, SNAI1, SNAI2, SUZ12, TNKS2, XIAP*
R-HSA-1433557	Signaling by SCF-KIT	21/600	14/507	21	14	0.007386588	p < 0.0001	p < 0.0001	Adult shift	*CBL, FYN, GRB10, GRB2, HRAS, JAK2, KIT, KITLG, KRAS, MMP9, NRAS, PIK3CA, PIK3R1, PIK3R2, PIK3R3, RAC1, SOCS1, STAT1, STAT3, STAT5B, YES1*	*CBL, CHEK1, FYN, GRB2, HRAS, KRAS, MMP9, NRAS, PIK3R1, PIK3R3, PRKCA, STAT1, STAT3, YES1*
R-HSA-9669938	Signaling by KIT in disease	15/600	10/507	15	10	0.005276134	p < 0.0001	p < 0.0001	Adult shift	*FYN, GRB2, HRAS, JAK2, KIT, KRAS, NRAS, PIK3CA, PIK3R1, PIK3R2, PIK3R3, STAT1, STAT3, STAT5B, YES1*	*FYN, GRB2, HRAS, KRAS, NRAS, PIK3R1, PIK3R3, STAT1, STAT3, YES1*

R-HSA-9670439	Signaling by phosphorylated juxtamembrane, extracellular and kinase domain KIT mutants	15/600	10/507	15	10	0.005276134	p < 0.0001	p < 0.0001	Adult shift	*FYN, GRB2, HRAS, JAK2, KIT, KRAS, NRAS, PIK3CA, PIK3R1, PIK3R2, PIK3R3, STAT1, STAT3, STAT5B, YES1*	*FYN, GRB2, HRAS, KRAS, NRAS, PIK3R1, PIK3R3, STAT1, STAT3, YES1*
R-HSA-9006925	Intracellular signaling by second messengers	56/600	54/507	56	54	-0.01317554	p < 0.0001	p < 0.0001	Fetal shift	*AGO2, AGO3, AKT1, AKT2, AKT3, BDNF, BMI1, CAMK4, CD28, CDKN1A, CDKN1B, CREB1, CSNK2A1, EGFR, ERBB2, ERBB3, ESR1, EZH2, FGF1, FGF10, FGF2, FGF20, FLT3, FOXO1, FRS2, FYN, GRB2, GSK3B, HDAC1, HDAC2, IRS1, IRS2, JUN, KIT, KITLG, MAPK1, MDM2, MET, MTA1, NTRK3, PDGFB, PHLPP1, PIK3CA, PIK3R1, PIK3R2, PIK3R3, PPP2CA, PREX2, PSMA3, PTEN, RAC1, RCOR1, SNAI1, SNAI2, SUZ12, TP53*	*ADCY3, AGO1, AGO2, AKT1, AKT2, BDNF, CAMK4, CD28, CDKN1A, CREB1, EGFR, ERBB2, ERBB3, ESR1, EZH2, FGF10, FGF2, FGF20, FGF8, FGFR1, FOXO1, FOXO3, FRS2, FYN, GRB2, GSK3B, HDAC1, HDAC2, HGF, IRAK1, IRS1, IRS2, LAMTOR5, MAPK1, MDM2, MET, MTA1, MTOR, MYD88, NTRK3, PDGFB, PDPK1, PIK3CD, PIK3R1, PIK3R3, PRKCA, PSME3, PTEN, RCOR1, SNAI1, SNAI2, SUZ12, TNKS2, XIAP*
R-HSA-199418	Negative regulation of the PI3K/AKT network	33/600	29/507	33	29	-0.00219921	p < 0.0001	p < 0.0001	Fetal shift	*AKT1, AKT2, AKT3, BDNF, CD28, EGFR, ERBB2, ERBB3, ESR1, FGF1, FGF10, FGF2, FGF20, FLT3, FRS2, FYN, GRB2, IRS1, IRS2, KIT, KITLG, MAPK1, MET, NTRK3, PDGFB, PHLPP1, PIK3CA, PIK3R1, PIK3R2, PIK3R3, PPP2CA, PTEN, RAC1*	*AKT1, AKT2, BDNF, CD28, EGFR, ERBB2, ERBB3, ESR1, FGF10, FGF2, FGF20, FGF8, FGFR1, FRS2, FYN, GRB2, HGF, IRAK1, IRS1, IRS2, MAPK1, MET, MYD88, NTRK3, PDGFB, PIK3CD, PIK3R1, PIK3R3, PTEN*
